# Tooth loss in adults: factors associated with the position and number of lost teeth

**DOI:** 10.11606/S1518-8787.2019053001318

**Published:** 2019-12-02

**Authors:** Valmir Vanderlei Gomes, Brunna Verna Castro Gondinho, Manoelito Ferreira Silva-Junior, Denise de Fátima Barros Cavalcante, Jaqueline Vilela Bulgareli, Maria da Luz Rosario de Sousa, Antonio Carlos Frias, Marília Jesus Batista, Antonio Carlos Pereira

**Affiliations:** I Universidade Estadual de Campinas. Programa de Pós-Graduação em Odontologia da Faculdade de Odontologia de Piracicaba. Piracicaba, SP, Brasil; II Universidade Estadual de Campinas. Departamento de Odontologia Social, área de Odontologia Preventiva e Saúde Pública da Faculdade de Odontologia de Piracicaba. Piracicaba, SP, Brasil; III Faculdade de Medicina de Jundiaí. Departamento de Saúde Coletiva. Jundiaí, SP, Brasil; IV Universidade de São Paulo. Faculdade de Odontologia. Departamento de Odontologia Social. São Paulo, SP, Brasil

**Keywords:** Adult, Tooth Loss, epidemiology, Risk Factors, Oral Health Surveys

## Abstract

**OBJECTIVE:**

To evaluate the factors associated with tooth loss in adults from the position and number of teeth lost in the dental arches.

**METHODS:**

This is a cross-sectional, population-based study with adults participating in the epidemiological survey of oral health of São Paulo in 2015. The outcome of the study was tooth loss, assessed by the proposed classification, namely: I) lost up to 12 back teeth; II) lost up to 12 teeth (including front teeth); and III) lost more than 12 teeth. A four-block analysis was conducted, supported by a conceptual theoretical model adapted for tooth loss. For the multinomial logistic regression, “individuals who did not lose teeth due to caries or periodontal disease” was used as reference (p < 0.05).

**RESULTS:**

Of 6,051 adults evaluated, 25.3% (n = 1,530) were classified in category I, 32.7% (n = 1,977) in II, 9.4% (n = 568) in III, and 1.9% (n = 117) were edentulous. Lower income and schooling, the perception of need for treatment and the last appointment motivated by routine, pain or extraction were associated with tooth loss, regardless of the classification. The negative evaluation of the dental service was associated with individuals who lost up to 12 teeth, both front and back. The presence of women and periodontal pocket were associated with tooth loss of up to 12 teeth, including front, and more than 12 teeth. Caries were associated with adults who lost up to 12 teeth, including front teeth.

**CONCLUSION:**

The proposed classification allowed the identification of differences between the associated factors. Thus, the need to consider such classification in future studies is evident.

## INTRODUCTION

In 2010, oral problems affected 3.9 billion people worldwide, with tooth loss being one of the 100 conditions that most affected the health of the world’s population in the last two decades. Tooth loss is the 36th most prevalent condition in the world, also being a public health problem^1^.

Given this context, tooth loss is an important marker of oral health due to representing the lack of care in the dental field, resulting from the increase in the severity level of the disease, the model of oral health care adopted, and the way individuals understand the disease^2^; thus, this condition tends to accumulate in the age range of adults^3^.

National surveys conducted in Brazil in 1986, 2003 and 2010 showed that the index of decayed, missing and filled teeth (DMFT) in adults was 22.5, 20.1 and 16.3, respectively, while the missing component was responsible for 65.4%, 65.7 % and 43.8% of occurrences^4^. The reduction of tooth loss in Brazilian adults in the last decade possibly indicates a combination of the reduction in the cohort effect of oral diseases^5^ and the improvement in socioeconomic conditions – especially education – and in the health system, such as exposure to fluoridation of water and use of fluoride dentifrices^4 , 6^, and the impact of the Brazilian Oral Health Policy, mainly due to the growth in the access to health services offered in the country.

The need for deeper studies on tooth loss becomes evident, not only considering the number^7^ but also the position that lost teeth occupy in the dental arch^8^. Individual social, economic and demographic characteristics are consistently associated with tooth loss in the literature^6^. According to Batista et al.^8^, older age and low social class were factors related to tooth loss; however, the use of categories of the new classification – which considers the position and number of lost teeth – allowed the identification of clinical conditions and behavioral factors such as the use of dental service and periodontal disease.

Such an understanding gains important projections when considering that the production of this information should guide the organization of health services and identify the factors that generate demands. This study, based on a new classification for tooth loss, aims to evaluate such losses considering the position and number of teeth lost in the dental arches and its associated factors in the adult population of the state of São Paulo.

## METHODS

### Study Design

This is a population-based and cross-sectional study with representativeness for six regions of the state of São Paulo (capital, metropolitan region and regional health departments II to XVII). Data for this study were taken from the 2015 epidemiological survey of oral health of the state of São Paulo (SBSP 2015)^9^.

### Ethical Aspects

This study was approved by the Research Ethics Committee of the School of Dentistry at Piracicaba from Universidade Estadual de Campinas (CEP-FOP/Unicamp), under no. 094/2015.

### Sample

The design of the sampling plan was prepared by conglomerate in two stages of drawing with probability proportional to population size (PPS), considering the sample weight and the design effect (deff) in each drawing stage. In the first stage, the state of São Paulo was stratified in six macroregions, the domains. For each domain, 33 municipalities were drawn and named primary sampling units; except for macroregion 1 (metropolitan region of the capital), for which 12 municipalities were drawn, in addition to the capital. In the second stage, two census tracts (census sampling unit) were drawn in each drawn municipality, also respecting the PPS on the tracts.

The sample size was defined based on frequency estimation, the variability to be investigated, and the acceptable margin of error. All these estimates come from the results of the *Pesquisa Nacional de Saúde Bucal de 2010* (SB Brasil 2010 – 2010 Brazilian Oral Health Survey)^10^ for the city of São Paulo (macroregion 1 – capital and metropolitan region) and countryside of the Southeast region (macroregions 2 to 6).

Considering the differences in oral health conditions in different age groups, the sample size was estimated for adults between 35 and 44 years old. The deff adopted was 2.0, with 8% margin of error and 95% confidence interval. The sample size for the age group between 35 and 44 years was 6,051.

### Data Collection

Clinical oral examinations were performed in the households visited, as advocated by the World Health Organization (WHO), with buccal plane mirrors, CPI probes and natural lighting, without previous drying or any type of prophylaxis procedure^11^. The minimum acceptable *kappa* value for each examiner, age group, and disease studied was 0.65^12^. The mean *kappa* value for periodontal disease was 0.76^9^, and for dental caries above 0.85.

Coronary dental caries, need for treatment, and periodontal condition were investigated as clinical conditions of oral health^11^. DMFT was used to evaluate the caries experience, resulting from the sum of the teeth affected by caries, missing and filled. The community periodontal index (CPI) was used to measure the presence of periodontal pockets.

Each volunteer responded to a questionnaire on demographics, socioeconomic factors and use of dental services. The SBSP 2015 questionnaire was answered via an interview at the time of the home examination.

### Variables

The dependent variable was classified into four categories based on the number of lost teeth and the position they occupied in the mouth, according to the classification of Batista et al.^8^: no tooth lost due to caries or periodontal disease; lost up to 12 back teeth; lost up to 12 teeth (including front teeth); and lost more than 12 teeth.

**Figure 1:**
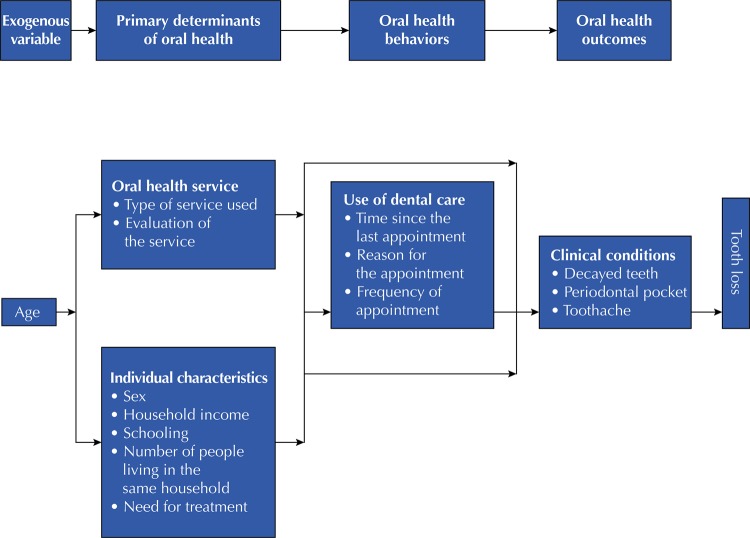
Theoretical conceptual model of tooth loss adapted for the study (Andersen & Davidson2). Epidemiological survey of oral health of the state of São Paulo, 2015.

The independent variables measured were related to baseline data and divided into four blocks ( Figure 1 ). The first block comprised one exogenous variable, age in years. In the second block, the main determinants of oral health were the type of dental service (public, private or insurance), evaluation of the service (good or not good), sex (female or male), family income (< R$ 1,500.00, R$ 1,500, 00–2,500, 00 or >R$ 2,500.00) and educational level (≤ 4 years, 5–10 years or ≥ 11 years of schooling), number of people living in the same household (≤ 3 people or ≥ 4 people) and need for treatment (yes or no). In the third block, on oral health behaviors, the use of dental service was evaluated as the time since the last visit to the dentist (< 1 year, 1–2 years or ≥ 3 years), and the reason for seeking dental services (routine, necessity, extraction, or pain). In the fourth block, the following oral health results were measured: decayed teeth (yes or no), periodontal pocket (< 4 mm or ≥ 4 mm) and toothache (yes or no).

### Data Analysis

The programs used to tabulate the data were the Statistical Package for the Social Sciences (SPSS), version 20.0, and Excel^®^(Microsoft Office). Absolute and percentage distribution, mean and standard deviation (SD) of the variables were obtained by descriptive analysis, in which the conditions of tooth loss were categorized according to the position and number of lost teeth.

Initially, bivariate analysis was conducted between the outcome (tooth loss) and the independent variables. The variables with p < 0.20 were used in the analysis, divided into four blocks, following the theoretical conceptual model “Aging, Ethnicity and Oral Health Outcomes” proposed by Andersen & Davidson^13^and adapted for tooth loss by Batista et al.^14 , 8^ ( Figure 1 ).

The variables were adjusted in each block, in which they were chosen to adjust the subsequent block (p < 0.20). The reference category of the analysis to perform the multinomial logistic regression (p < 0.05) were individuals who had not lost any teeth due to the presence of caries or periodontal disease.

## RESULTS

In total, 6,051 adults aged between 35 to 44 years were examined, representing the adult population living in the state of São Paulo. Table 1 shows the sample’s demographic and socioeconomic characteristics. Most of them were women, with family income lower than R$ 1,500.00, with nine or more years of schooling, and living with more than four people. The mean DMFT was 15.84 (SD = 16.29) teeth with caries experience, 1.53 (SD = 1.74) decayed teeth, 6.30 (SD = 6.79) missing teeth, and 7.46 (SD = 7.87) filled teeth.

**Table 1:** Demographic and socioeconomic characteristics and health care practices of adults aged 35 to 44 years living in the state of São Paulo in 2015.

Variables	n	%
*Exogenous variables*		
*Determinants of oral health*		
Type of service used (n = 5,709)		
Public	2,288	40.1
Insurance	575	10.5
Private	2,846	49.3
Evaluation of the service used (n = 5,736)		
Not good	817	15.8
Good	4,919	84.2
Sex (n = 6,051)		
Female	4,108	69.6
Male	1,943	30.4
Schooling (n = 5,653)		
< 5 years	840	14.7
5–9 years	1,630	30.3
> 9 years	3,183	55.0
Household income (n = 5,309)		
< R$ 1,500.00	2,224	46.4
Between R$ 1,500.00 and R$ 2,500.00	1,792	31.1
> R$ 2,500.00	1,293	22.4
Number of people living in the same house (n = 5,883)		
Up to 3 people	2,431	40.2
More than 4 people	3,452	59.8
Perception of the need for treatment (n = 4,721)		
Yes	4,599	81.2
No	122	18.8
*Oral health behaviors*		
Reason for the appointment (n = 4,326)		
Routine	133	23.0
Pain	1,046	18.9
Extraction	617	11.0
Treatment	2,530	47.1
Time since the last appointment (n = 5,694)		
< 1 year	3,185	52.2
1–2 years	1,473	26.6
> 2 years	1,036	21.2
*Clinical conditions of oral health*		
Decayed teeth (n = 6,051)		
Yes	3,306	57.2
No	2,745	42.8
Periodontal pocket (n = 5,859)		
Yes	4,332	72.9
No	1,527	27.1
Pain (n = 5,219)		
Yes	1,824	32.0
No	3,395	68.0

Note: Some variables do not total 6,051 participants due to lost data.

The percentage of adults who had not lost any teeth due to oral diseases was 24.8% (1,500). Table 2 describes the frequency of tooth loss according to the proposed classification. A total of 5.9% (n = 359) of the adults examined presented loss of 1 to 4 first molars, 25.3% (n = 1,530) lost up to 12 back teeth, 32.7% (n = 1,977) lost up to 12 teeth, including one or more front teeth, 9.4% (n = 568) lost from 13 to 31 teeth, and 1.9% (n = 117) were edentulous.

**Table 2:** Distribution according to the classification of tooth loss in adults living in the state of São Paulo, Brazil. Epidemiological survey of oral health of the state of São Paulo, 2015.

Classification of tooth loss*	n	%
Did not present tooth loss	1,500	24.8
Lost up to 12 back teeth	1,889	31.2
Lost up to 12 teeth, including front ones	1,977	32.7
Lost more than 12 teeth	685	11.3

*Due to caries or periodontal disease.

Table 3 shows the results of the bivariate analyses and the crude odds ratio for the new classification of tooth loss. Table 4 presents the adjusted data according to the classification of tooth loss. The loss of up to 12 front teeth was associated with women (OR = 1.11; 95%CI 0.94–1.31), income lower than R$ 1,500.00 (OR = 1.39; 95%CI 1.13–1.71) and between R$ 1,500.00 and 2,500.00 (OR = 1.78; 95%CI 1.46–2.17), schooling between 5 and 9 years of study (OR = 1.39; 95%CI 1.14–1.70) and demand for service motivated by routine (OR = 1.30; 95%CI 1.08–1.56), pain (OR = 2.04; 95%CI 1.43–2.90) and for tooth extraction (OR = 1.37; 95%CI 1.07 – 1.75). For those who lost up to 12 teeth, including front ones, the associated factors were women (OR = 1.19; 95%CI 1.00–1.42), income lower than R$ 1,500.00 (OR = 1.65; 95%CI 1.33–2.05) and between R$ 1,500.00 and 2,500.00 (OR = 1.82; 95%CI 1.47–2.25), schooling less than 5 years (OR = 2.2; 95%CI 1.68–2.90) and between 5 and 9 years (OR = 2.01; 95%CI 1.65–2.45), and positive perception about the need for treatment (OR = 1.95; 95%CI 1.57–2.43), in addition to demand for service motivated by routine (OR = 1.83; 95%CI 1.50–2.24), pain (OR = 3.49; 95%CI 2.46–4.97) and for tooth extraction (OR = 1.97; 95%CI 1.53–2.55). For those who lost more than 12 teeth, women (OR = 1.42; 95%CI 1.09–1.86), income lower than R$ 1,500.00 (OR = 1.63; 95%CI 1.13–2.33) and between R$ 1,500.00 and 2,500.00 (OR = 1.97; 95%CI 1.37–2.83), schooling less than 5 years (OR = 7.19; 95%CI 5.02–10.32) and between 5 and 9 years (OR = 4.71; 95%CI 3.49–6.37) and demand for service motivated by routine (OR = 4.09; 95%CI 2.70–6.17), pain (OR = 11.79; 95%CI 7.02–19.80) and tooth extraction (OR = 3.60; 95%CI 2.25–5.77).

**Table 3:** Crude analysis of the associated factors according to the classification of tooth loss in adults aged 35 to 44 years. Epidemiological survey of oral health of the state of São Paulo, 2015.

Variables		Classification of tooth loss								
		Lost up to 12 back teeth			Lost up to 12 teeth, including front ones			Lost more than 12 teeth		
		Crude OR	95%CI	p-value	Crude OR	95%CI	p-value	Crude OR	95%CI	p-value
Primary determinants of health										
Sex	Female	1.14	0.99–1.32	0.016	1.28	1.11–1.47	0.001	1.46	1.20–1.78	< 0.001
	Male	1.00			1.00			1.00		
Household income	Low	1.68	1.40–2.01	< 0.001	2.71	2.25–3.24	< 0.001	4.31	3.25–5.71	< 0.001
	Mean	1.86	1.55–2.23	< 0.001	2.22	1.83–2.68	< 0.001	2.81	2.08–3.78	< 0.001
	High	1.00			1.00			1.00		
Schooling	< 5 years	1.54	1.20–1.98	0.001	3.25	2.56–4.11	< 0.001	13.22	9.91–17.63	< 0.001
	5–9 years	1.57	1.32–1.87	< 0.001	2.64	2.23–3.13	< 0.001	6.44	5.05–8.23	< 0.001
	> 9 years	1.00			1.00			1.00		
Number of people living in the same household	< 3 people	1.18	1.03–1.36	0.018	1.26	1.10–1.45	0.001	1.20	0.99–1.45	0.052
	> 3 people	1.00			1.00			1.00		
Perception of the need for treatment	No	1.69	1.45–1.98	< 0.001	3.02	2.54–3.58	< 0.001	1.96	1.56–2.46	< 0.001
	Yes	1.00			1.00			1.00		
Type of service	Public	1.4	1.20–1.63	< 0.001	1.44	1.24–1.66	< 0.001	1.77	1.45–2.15	< 0.001
	Insurance	1.33	1.05–1.68	0.015	1.33	1.05–1.68	0.497	0.67	0.46–1.0	0.051
	Private	1.00			1.00			1.00		
Evaluation of treatment	Not good	1.57	1.26–1.96	< 0.001	1.75	1.41–2.16	< 0.001	1.79	1.36–2.35	< 0.001
	Good	1.00			1.00			1.00		
Oral health behaviors										
Time since the last appointment	< 1 year	1.28	1.04–1.56	0.016	1.48	1.21–1.80	< 0.001	2.84	2.23–3.60	< 0.001
	1–2 years	1.19	1.01–1.41	0.034	1.24	1.05–1.46	0.011	1.28	1.01–1.62	0.039
	> 2 years	1.00			1.00			1.00		
Reason for the appointment	Routine	1.55	1.32–1.83	< 0.001	2.48	2.08–2.95	< 0.001	5.08	3.68–7.00	< 0.001
	Pain	2.41	1.78–3.26	< 0.001	5.11	3.81–6.88	< 0.001	21.55	14.45–32.13	< 0.001
	Extraction	1.88	1.52–2.34	< 0.001	3.39	2.72–4.22	< 0.001	6.34	4.40–9.16	< 0.001
	Treatment	1.00			1.00			1.00		
Oral health outcomes										
Periodontal pocket	Yes	1.24	1.05–1.47	0.012	1.96	1.67–2.30	< 0.001	2.44	1.96–3.03	< 0.001
	No	1.00			1.00			1.00		
Dental caries	Yes	1.31	1.14–1.50	< 0.001	1.97	1.69–2.30	< 0.001	1.82	1.49–2.24	< 0.001
	No	1			1			1.00		
Toothache	Yes	1.32	1.12–1.55	0.001	1.97	1.69–2.30	< 0.001	1.83	1.49–2.24	< 0.001
	No	1.00			1.00			1.00		

**Table 4:** Crude analysis of the associated factors according to the classification of tooth loss in adults aged 35 to 44 years. Epidemiological survey of oral health of the state of São Paulo, 2015.

Variables		Adjusted OR	95%CI	p-value
Lost up to 12 teeth				
Sex				
	Female	1.11	0.94–1.31	0.204
	Male	1.00		
Income	< R$ 1,500.00	1.39	1.13–1.71	0.002
	R$ 1,500.00–2,500.00	1.78	1.46–2.17	< 0.001
	> R$ 2,500.00	1.00		
Schooling	< 5 years	1.28	0.96–1.69	0.088
	5–9 years	1.39	1.14–1.70	0.001
	> 9 years	1.00		
Perception of the need for treatment	Yes	1.36	1.12–1.65	0.002
	No	1.00		
Reason for the appointment	Routine	1.30	1.08–1.56	0.006
	Pain	2.04	1.43–2.90	< 0.001
	Extraction	1.37	1.07–1.75	0.013
	Treatment	1.00		
Evaluation of the service	Good	1.33	1.04–1.70	0.024
	Not good	1.00		
Type of service	Public	1.17	0.98–1.39	0.080
	Insurance	1.39	1.07–1.80	0.012
	Private	1.00		
Periodontal pocket	Yes	1.05	0.86–1.28	0.652
	No	1.00		
Dental caries	Yes	1.01	0.85–1.20	0.878
	No	1.00		
Lost up to 12 teeth, including front ones				
Sex	Female	1.19	1.00–1.42	0.044
	Male	1.00		
Income	< R$ 1,500.00	1.65	1.33–2.05	< 0.001
	R$ 1,500.00–2,500.00	1.82	1.47–2.25	< 0.001
	> R$ 2,500.00	1.00		
Schooling	< 5 years	2.21	1.68–2.90	< 0.001
	5–9 years	2.01	1.65–2.45	< 0.001
	> 9 years	1.00		
Perception of the need for treatment	Yes	1.95	1.57–2.43	< 0.001
	No	1.00		
Reason for the appointment	Routine	1.83	1.50–2.24	< 0.001
	Pain	3.49	2.46–4.97	< 0.001
	Extraction	1.97	1.53–2.55	< 0.001
	Treatment	1.00		
Evaluation of treatment	Good	1.30	1.02–1.67	0.036
	Not good	1.00		
Type of service	Public	0.97	0.81–1.16	0.764
	Insurance	1.17	0.89–1.54	0.269
	Private	1.00		
Periodontal pocket	Yes	1.33	1.10–1.62	0.004
	No	1.00		
Dental caries	Yes	1.35	1.13–1.61	0.001
	No	1.00		
Lost more than 12 teeth				
Sex	Female	1.42	1.09–1.86	0.010
	Male	1.00		
Income	< R$ 1,500.00	1.63	1.13–2.33	0.008
	R$ 1,500.00–2,500.00	1.97	1.37–2.83	< 0.001
	> R$ 2,500.00	1.00		
Schooling	< 5 years	7.20	5.02–10.32	< 0.001
	5–9 years	4.71	3.49–6.37	< 0.001
	> 9 years	1.00		
Perception of the need for treatment	Yes	1.81	1.25–2.60	0.001
	No	1.00		
Reason for the appointment	Routine	4.09	2.70–6.17	< 0.001
	Pain	11.79	7.02–19.80	< 0.001
	Extraction	3.60	2.25–5.77	< 0.001
	Treatment	1.00		
Evaluation of the service	Good	1.12	0.78–1.61	0.521
	Not good	1.00		
Type of service	Public	0.96	0.73–1.24	0.733
	Insurance	0.75	0.45–1.24	0.266
	Private	1.00		
Periodontal pocket	Yes	1.44	1.10–1.90	0.009
	No	1.00		
Dental caries	Yes	1.15	0.88–1.51	0.296
	No	1.00		

Note: The reference category for the multinomial regression analysis was “not having lost any tooth due to caries or periodontal disease”.

## DISCUSSION

In this study, lower income and schooling, the perception of the need for treatment, and last appointment motivated by routine, pain or extraction were associated with tooth loss, regardless of the classification. The negative evaluation of the dental service was associated with individuals who lost up to 12 teeth, both front and back. The presence of women and periodontal pocket were associated with tooth loss of up to 12 teeth, including front, and of more than 12 teeth. Caries were associated only with individuals who lost up to 12 teeth, including front ones.

The literature presents studies that numerically assess tooth loss using the presence of 20 teeth or more as the cutoff base^7^. We verified that the use of the classification of tooth loss proposed by Batista et al.^8^ was able to measure associated factors more specifically according to the number and position of teeth in the dental arch. This classification is based on the reduced dental arch theory, which considers as satisfactory the presence of ten pairs of occlusive teeth without aesthetic gaps^15^ and considers losses due to dental caries and periodontal disease, excluding teeth deemed to be absent congenitally or due to orthodontic reasons, which was already a WHO criterion^11^. Clinically, the incorporation of teeth lost due to periodontal disease by this new classification allows the identification of the aesthetic and functional issue, which are also important for the planning of the oral rehabilitation of patients. Moreover, a previous study identified that the position and number of lost teeth have different affects the quality of life of adults^7^.

Several studies have associated tooth loss with lower income and schooling^16 , 17 , 18 , 6 , 19^. This can be explained by the fact that poorer and less educated individuals live in places with lower coverage of fluoridation of water^20^, have impaired access to dental services^21 , 4^ and hygiene products^4^, and practice inappropriate habits such as consuming more sugar^22^ and brushing the teeth less frequently^23^.

Data from the National Household Sample Survey (PNAD) showed that individuals with higher schooling present higher frequency in dental appointments: 67.4% among those with complete higher education and 36.6% among individuals without any schooling or with incomplete elementary education^24^. For Chrysanthakopoulos^25^, schooling also has affected the self-perception of individuals about the state of their oral health condition and the assessment of the need for dental treatment. In this study, an association was found between higher schooling level and lower number of lost teeth; however, the perception of the need for treatment was also associated with tooth loss, regardless of the classification. The study by Santillo et al.^26^ in a rural population of Pernambuco (PE) found a relationship between tooth loss and self-perceived negative oral health. These results indicate that the dentistry model still has a mutilation character and that the perception of the need for treatment occurs only in advanced stages of oral diseases, thus determining the late search for dental services^27 , 28^.

This idea can be reinforced in this study, in which the last dental appointment motivated by routine, pain or extraction was associated with tooth loss, regardless of the classification. Although the literature relates the demand for dental services due to pain with the prevalence^8 , 29^ and incidence of tooth loss^5^, this is probably the first study that also shows association with the search for the service motivated by tooth extraction and routine. This result can be explained by the history of Brazilian oral health care – especially among adults –, which is marked by the restriction of access and high demand^4^.

Moreover, the late search for oral health services determines the progression of oral diseases, resulting in the need for mutilating procedures and techniques and especially tooth loss^6 , 28 , 30^. Another relevant aspect is how to turn the search for dental services into a routine for an economically active population. The study by Silva-Junior et al.^28^ found that the choice for extracting teeth rather than keeping them is mainly due to the absence of another treatment option at the time of appointment, and to the high cost of the procedures necessary to maintain teeth.

Another relevant aspect for our study was the association between the classification of tooth loss and the evaluation of dental services. The evaluation of health services by patients assists in the construction of indicators aiming at the implementation of health strategies of the service, guiding the actions of prevention and promotion^31^. A study conducted in Bahia found inequality in the use of oral health services even among patients of the public service at different care levels. Those who had lower schooling and who were exposed to a worse service organization did not use the service as often^32^.

When compared with private supplementary care, the public service presented a profile of vulnerability, although its disorganization does not favor its use. The results reinforce that coping with inequalities in the access and use of public health services is dependent on how the local government plans the project and its ability to reorganize dental care. Therefore, we must think about ways to promote the use and satisfaction with public health services, especially among adults. This economically active age group faces the restriction of the working hours of health units, hindering their access to health care and, consequently, to the management of the initial stages of the main oral diseases.

In this study, association was found between the clinical conditions of oral health, caries and periodontal disease, with tooth loss among individuals who have a greater number of back teeth. From this finding, we can infer that the presence of back teeth increases the occurrence of caries and periodontal disease, which would explain the maintenance of this association even in the final adjusted model. Back teeth are the most affected by oral diseases^5^, mainly due to the lack of knowledge of the presence of permanent teeth still in childhood and for being in non-aesthetic areas, making it difficult to visualize the need for treatment in the absence of painful symptomatology and delaying the demand for dental service for treatment.

This can be evidenced in our study since the variable of self-perception of the need for treatment was associated with the three tooth loss categories. Individuals who lost up to 12 teeth, including front ones, and thus still maintain back teeth, were associated with the presence of caries and periodontal pocket, and for losses of more than 12 teeth, only to periodontal pocket. The study by Batista et al.^8^ also found an association between individuals who lost 12 teeth, including front ones, with periodontal pocket. Therefore, studies that consider the position of the lost teeth can infer more reliably the association of variables for tooth loss.

Dannewitz et al.^33^ showed that access to periodontal therapy results in a good prognosis of molars. According to the results shown in our study, the presence of periodontal pocket was not associated with participants who already lost back teeth. The scarcity of access to specialized services to perform periodontal treatment can contribute to the large number of tooth losses, mainly of the molars. Despite the launch, in 2004, of the current Brazilian Oral Health Policy and the consequent expansion of the supply of specialized dental services, the number of periodontal and endodontic procedures did not increase in all studied municipalities. Several possibilities and justifications can explain this, such as the availability of the workforce and their geographic distribution, the ease or not of access to dental services, the characteristics of the service administration, or the organization of the care network and of the work process^34^. We must stress that the policy is still recent, especially the secondary care actions.

In this study, the main component identified in the caries experience of adults was ‘restored teeth.’ This datum was also verified in the last national oral health survey for the south and southeast regions of the country, both of which present better socioeconomic conditions and may reflect in a better case management in the early stages of dental caries and incorporation of less invasive treatments^4^, as well as in the impact of the insertion and expansion of the Brazilian Oral Health Policy. The diagnosis of oral health conditions and of the population’s treatment needs, and the evaluation of the current health care model, is crucial as a first step towards the scheduling and planning in oral health, enabling the establishment of priorities for action and resource allocation to improve the health conditions of the population^35^.

The limitations of the study are in its cross-sectional nature, with exposure and outcome evaluated at a single moment in time, and in the possibility of prevalence bias; the greater participation of women given that it was a household survey with adults is another limitation. Moreover, the questionnaire included past experiences of dental care, which depend on the individual’s memory for accuracy.

The categories of the new tooth loss classification, considering the position and number of lost teeth, allowed different associated factors to be identified. The results of our study indicate an evident need to consider a qualitative and quantitative assessment of tooth loss, so such an occurrence is not underestimated. This is a fundamental observation to be considered in future studies, including to subsidize decision making in the supply and organization of dental services.
