# Supplementary material for: Tooth loss in adults: factors associated with the position and number of lost teeth
Source: Rev Saude Publica. 2019 Dec 2;53:105. doi: 10.11606/S1518-8787.2019053001318 (PMC6904121; doi:10.11606/S1518-8787.2019053001318)
Supplement: Translation

# Translation: Perdas dentárias em adultos: fatores associados à posição e ao número de dentes perdidos

**Authors:** Valmir Vanderlei Gomes, Brunna Verna Castro Gondinho, Manoelito Ferreira Silva-Junior, Denise de Fátima Barros Cavalcante, Jaqueline Vilela Bulgareli, Maria da Luz Rosario de Sousa, Antonio Carlos Frias, Marília Jesus Batista, Antonio Carlos Pereira

**Affiliations:** I Universidade Estadual de Campinas. Programa de Pós-Graduação em Odontologia da Faculdade de Odontologia de Piracicaba. Piracicaba, SP, Brasil; II Universidade Estadual de Campinas. Departamento de Odontologia Social, área de Odontologia Preventiva e Saúde Pública da Faculdade de Odontologia de Piracicaba. Piracicaba, SP, Brasil; III Faculdade de Medicina de Jundiaí. Departamento de Saúde Coletiva. Jundiaí, SP, Brasil; IV Universidade de São Paulo. Faculdade de Odontologia. Departamento de Odontologia Social. São Paulo, SP, Brasil

**Keywords:** Adulto, Perda de Dente, epidemiologia, Fatores de Risco, Inquéritos de Saúde Bucal

## Abstract

**OBJETIVO:**

Avaliar os fatores associados à perda dentária em adultos a partir da posição e do número de dentes perdidos nos arcos dentários.

**MÉTODOS:**

Estudo transversal, populacional, com adultos participantes do levantamento epidemiológico de saúde bucal de São Paulo em 2015. O desfecho do estudo foi a perda dentária, avaliada pela classificação proposta, a saber: I) perdeu até 12 dentes posteriores; II) perdeu até 12 dentes (incluindo dentes anteriores); e III) perdeu acima de 12 dentes. Realizou-se uma análise em quatro blocos, apoiada em um modelo teórico conceitual adaptado para perdas dentárias. Para a regressão logística multinominal, usou-se “indivíduos que não tenham perdido dentes por cárie ou doença periodontal” como referência (p < 0,05).

**RESULTADOS:**

De 6.051 adultos avaliados, 25,3% (n = 1.530) foram classificados na categoria I, 32,7% (n = 1.977) na II, 9,4% (n = 568) na III e 1,9% (n = 117) eram desdentados totais. A menor renda e escolaridade, a percepção de necessidade de tratamento e última consulta motivada por rotina, dor ou extração foram associados à perda dentária, independentemente da classificação. A avaliação negativa do serviço odontológico esteve associada aos indivíduos que perderam até 12 dentes, tanto anteriores quanto posteriores. O sexo feminino e a presença de bolsa periodontal foram associados a perdas dentárias de até 12 dentes, incluindo anteriores, e acima de 12 dentes. A presença de cárie foi associada aos adultos que perderam até 12 dentes, incluindo dentes anteriores.

**CONCLUSÃO:**

A classificação proposta permitiu a identificação de diferenças entre os fatores associados. Assim, fica evidente a necessidade de considerá-la em estudos futuros.

## INTRODUÇÃO

Em 2010, os problemas bucais afetaram 3,9 bilhões de pessoas no mundo, sendo a perda dentária uma entre as cem condições que mais impactaram a saúde da população mundial nas últimas duas décadas. Ela é a 36ª condição mais prevalente no mundo, evidenciando-se ainda como um problema de saúde pública^1^.

Nesse contexto, a perda dentária é um importante marcador de saúde bucal, pois representa o descaso no âmbito odontológico, resultando do aumento do nível de gravidade da doença, do modelo de atenção à saúde bucal adotado e da forma como os indivíduos compreendem o agravo^2^; por isso, tende a acumular-se na faixa etária de adultos^3^.

Levantamentos nacionais realizados no Brasil em 1986, 2003 e 2010 revelaram que o índice de dentes permanentes cariados, perdidos e obturados (CPOD) em adultos foi de 22,5, 20,1 e 16,3, respectivamente, enquanto o componente perdido foi responsável por 65,4%, 65,7% e 43,8% das ocorrências^4^. A redução das perdas dentárias em adultos brasileiros na última década indica, possivelmente, uma combinação da redução no efeito coorte das doenças bucais^5^e da melhora das condições socioeconômicas, em especial da educação, e do sistema de saúde, como a exposição à fluoretação de águas e uso de dentifrícios fluoretados^4 , 6^, além do impacto da Política Nacional de Saúde Bucal, principalmente pelo aumento do acesso aos serviços de saúde bucal ofertados no país.

Sendo assim, fica evidente a necessidade de se estudar de forma mais aprofundada o impacto da perda dentária, não só pelo número^7^, mas também pela posição que os dentes perdidos ocupam na arcada dentária^8^. As características sociais, econômicas e demográficas individuais são associadas às perdas dentárias de maneira consistente na literatura^6^. Segundo Batista et al.^8^, maior idade e baixa classe social foram fatores relacionados à perda dentária, porém a utilização de categorias da nova classificação, que considera a posição e o número de dentes perdidos, permitiram identificar condições clínicas e fatores comportamentais, como a utilização de serviço odontológico e a doença periodontal.

Tal entendimento ganha importantes projeções, uma vez que a produção dessas informações deve orientar a organização dos serviços de saúde e identificar os fatores que geram tais demandas. Este estudo, baseado em uma nova classificação para perdas dentárias, tem como objetivo avaliar tais perdas, considerando a posição e o número de dentes perdidos nos arcos dentários, e seus fatores associados na população de adultos do estado de São Paulo.

## MÉTODOS

### Delineamento do Estudo

O estudo é do tipo transversal, de base populacional, com representatividade para seis regiões do estado de São Paulo (capital, região metropolitana e os departamentos regionais de saúde II a XVII). Os dados desse estudo foram retirados do levantamento epidemiológico de saúde bucal do estado de São Paulo de 2015 (SBSP 2015)^9^.

### Aspectos Éticos

O estudo foi aprovado pelo Comitê de Ética em Pesquisa da Faculdade de Odontologia de Piracicaba da Universidade Estadual de Campinas (CEP-FOP/Unicamp), sob o número 094/2015.

### Amostra

O delineamento do plano amostral foi elaborado por conglomerado em dois estágios de sorteio com probabilidade proporcional ao tamanho da população (PPT), levando em consideração o peso amostral e o efeito do desenho ( *deff* ) em cada etapa de sorteio. Na primeira, o estado de São Paulo foi estratificado em seis macrorregiões, os domínios. De cada domínio foram sorteados 33 municípios, que foram denominados unidades primárias de amostragem, com exceção da macrorregião 1 (região metropolitana da capital) onde foram sorteados 12 municípios, além da capital. Na segunda etapa foram sorteados dois setores censitários (unidade censitária de amostragem) em cada município sorteado, também respeitando a PPT nos setores.

O tamanho da amostra foi definido com base na estimativa de frequência, variabilidade a ser investigada e margem de erro aceitável. Todas essas estimativas provêm dos resultados da Pesquisa Nacional de Saúde Bucal de 2010 (SB Brasil 2010)^10^referentes à cidade de São Paulo (macrorregião 1 – capital e região metropolitana) e interior do Sudeste (para as macrorregiões 2 a 6).

Considerando as diferenças das condições de saúde bucal nas diferentes faixas etárias, o tamanho amostral foi estimado para adultos de 35 a 44 anos. Adotou-se um *deff* de 2,0, margem de erro de 8% e intervalo de confiança de 95%. O tamanho amostral para a faixa etária de 35 a 44 anos foi de 6.051.

### Coleta de Dados

Nos domicílios visitados, realizaram-se os exames bucais clínicos, conforme preconiza a Organização Mundial de Saúde (OMS), com espelhos planos bucais, sondas CPI e iluminação natural, sem que houvesse secagem prévia ou qualquer tipo de profilaxia^11^. O valor mínimo aceitável de *kappa* para cada examinador, grupo etário e agravo estudado foi igual a 0,65^12^. Os valores médios de *kappa* para a doença periodontal foi de 0,76^9^e de cárie dentária, acima de 0,85.

Cárie dentária coronária, necessidade de tratamento e condição periodontal foram pesquisadas como condições clínicas de saúde bucal^11^. O CPOD foi utilizado para avaliar a experiência de cárie, resultante da soma dos dentes acometidos por cárie, perdidos e obturados. Utilizou-se o índice periodontal comunitário (CPI) para mensurar a presença de bolsa periodontal.

Cada voluntário respondeu a um questionário para verificação de fatores demográficos, socioeconômicos e de utilização de serviço odontológico. O questionário do SBSP 2015 foi respondido por meio de entrevista no momento da realização do exame domiciliar.

### Variáveis

A variável dependente foi classificada em quatro categorias baseadas no número de dentes perdidos e na posição que ocupavam na boca, conforme a classificação de Batista et al.^8^: não perdeu nenhum dente por cárie ou doença periodontal; perdeu até 12 dentes posteriores; perdeu até 12 dentes (incluindo dentes anteriores); e perdeu acima de 12 dentes.

As variáveis independentes medidas foram relacionadas aos dados de linha de base e divididas em quatro blocos ( Figura 1 ). No primeiro, uma variável exógena era a idade em anos. No segundo bloco, os principais determinantes da saúde bucal foram o tipo de serviço odontológico (público, privado ou convênio), avaliação do serviço (bom ou não bom), sexo (feminino ou masculino), renda familiar (< R$ 1.500,00, R$ 1.500,00–2.500,00 ou > R$ 2.500,00) e nível de escolaridade (≤ 4 anos, 5–10 anos ou ≥ 11 anos de estudo), número de pessoas que vivem no mesmo domicílio (≤ 3 pessoas ou ≥ 4 pessoas) e necessidade de tratamento (sim ou não). No terceiro bloco, sobre comportamentos de saúde bucal, a utilização do serviço odontológico foi avaliada como o tempo desde a última visita ao dentista (< 1 ano, 1–2 anos ou ≥ 3 anos) e motivo de busca de serviços odontológicos (rotina, necessidade, extração ou dor). No quarto bloco, aferiram-se os resultados da saúde bucal: dentes decaídos (sim ou não), bolsa periodontal (< 4 mm ou ≥ 4 mm) e dor dental (sim ou não).

**Figura 1:** Modelo conceitual teórico das perdas dentárias adaptado para o estudo (Andersen e Davidson2). Levantamento epidemiológico de saúde bucal do estado de São Paulo, 2015.

### Análise dos Dados

Utilizou-se o programa *Statistical Package for the Social Sciences* (SPSS), versão 20.0, e o Excel^®^(Microsoft Office) para tabular os dados. Obtiveram-se a distribuição absoluta e percentual, média e desvio-padrão (DP) das variáveis por uma análise descritiva, na qual as condições de perdas dentárias foram categorizadas conforme posição e número de dentes perdidos.

Inicialmente foi feita a análise bivariada entre o desfecho (perda dentária) e as variáveis independentes. As variáveis com p < 0,20 foram utilizadas na análise, dividida em quatro blocos, seguindo o modelo conceitual teórico “ *Aging, Ethnicity and Oral Health Outcomes* ” proposto por Andersen e Davidson^13^e adaptado para perdas dentárias por Batista et al.^14 , 8^( Figura 1 ).

As variáveis foram ajustadas em cada bloco, no qual eram escolhidas para ajustar o bloco subsequente (p < 0,20). A categoria de referência da análise para realizar a regressão logística multinominal (p < 0,05) foram os indivíduos que não tinham perdido nenhum dente por presença de cárie ou doença periodontal.

## RESULTADOS

Um total de 6.051 adultos entre 35 a 44 anos foi examinado, representando a população de adultos residentes no estado de São Paulo. A Tabela 1 apresenta suas características demográficas e socioeconômicas. A maioria dos examinados foi de mulheres, com renda familiar menor que R$ 1.500,00, com nove ou mais anos de estudo e que residem com mais de quatro pessoas. A média do CPOD foi de 15,84 (DP = 16,29) dentes com experiência de cárie, sendo 1,53 (DP = 1,74) dentes cariados, 6,30 (DP = 6,79) dentes perdidos e 7,46 (DP = 7,87) dentes restaurados.

**Tabela 1:** Características demográficas, socioeconômicas e de práticas em saúde de adultos entre 35 e 44 anos residentes no estado de São Paulo em 2015.

Variáveis	n	%
*Variáveis exógenas*		
*Determinantes de saúde bucal*		
Tipo de serviço utilizado (n = 5.709)		
Público	2.288	40,1
Convênio	575	10,5
Particular	2.846	49,3
Avaliação do serviço utilizado (n = 5.736)		
Não boa	817	15,8
Boa	4.919	84,2
Sexo (n = 6.051)		
Feminino	4.108	69,6
Masculino	1.943	30,4
Escolaridade (n = 5.653)		
< 5 anos	840	14,7
5–9 anos	1.630	30,3
> 9 anos	3.183	55,0
Renda familiar (n = 5.309)		
< R$ 1.500,00	2.224	46,4
Entre R$ 1.500,00 e R$ 2.500,00	1.792	31,1
> R$ 2.500,00	1.293	22,4
Número de pessoas morando na mesma casa (n = 5.883)		
Até 3 pessoas	2.431	40,2
Mais de 4 pessoas	3.452	59,8
Percepção da necessidade de tratamento (n = 4.721)		
Sim	4.599	81,2
Não	122	18,8
*Comportamentos em saúde bucal*		
Motivo da consulta (n = 4.326)		
Rotina	133	23,0
Dor	1.046	18,9
Extração	617	11,0
Tratamento	2.530	47,1
Tempo desde a última consulta (n = 5.694)		
< 1 ano	3.185	52,2
1–2 anos	1.473	26,6
> 2 anos	1.036	21,2
*Condições clínicas de saúde bucal*		
Dentes cariados (n = 6.051)		
Sim	3.306	57,2
Não	2.745	42,8
Bolsa periodontal (n = 5.859)		
Sim	4.332	72,9
Não	1.527	27,1
Dor (n = 5.219)		
Sim	1.824	32,0
Não	3.395	68,0

Nota: Algumas variáveis não totalizam 6.051 participantes por apresentar dados perdidos.

Um total de 24,8% (n = 1.500) dos adultos não havia perdido nenhum dente por doenças bucais. A frequência de perdas dentárias segundo a classificação proposta está descrita na Tabela 2 . Um total de 5,9% (n = 359) dos adultos examinados apresentava perda de 1 a 4 primeiros molares, 25,3% (n = 1.530) perderam até 12 dentes posteriores, 32,7% (n = 1.977) perderam até 12 dentes, incluindo um ou mais dentes anteriores, 9,4% (n = 568) perderam de 13 a 31 dentes, e 1,9% (n = 117) eram desdentados totais.

**Tabela 2:** Distribuição segundo a classificação da perda dentária em adultos residentes no estado de São Paulo, Brasil. Levantamento epidemiológico de saúde bucal do estado de São Paulo, 2015.

Classificação das perdas dentárias*	n	%
Não apresentou perda dentária	1.500	24,8
Perdeu até 12 dentes posteriores	1.889	31,2
Perdeu até 12 dentes, incluindo anteriores	1.977	32,7
Perdeu acima de 12 dentes	685	11,3

*Por cárie ou doença periodontal.

Na Tabela 3 são apresentados os resultados das análises bivariadas e a razão de chances bruta para a nova classificação das perdas dentárias. Na Tabela 4 constam os dados ajustados segundo a classificação das perdas dentárias. A perda de até 12 dentes anteriores foi associada com o sexo feminino (OR = 1,11; IC95% 0,94–1,31), renda menor que R$ 1.500,00 (OR = 1,39; IC95% 1,13–1,71) e entre R$ 1.500,00 e 2.500,00 (OR = 1,78; IC95% 1,46–2,17), escolaridade entre 5 e 9 anos de estudo (OR = 1,39; IC95% 1,14–1,70) e a procura do serviço por rotina (OR = 1,30; IC95% 1,08–1,56), motivada por dor (OR = 2,04; IC95% 1,43–2,90) e para extração dentária (OR = 1,37; IC95% 1,07–1,75). Para os que perderam até 12 dentes, incluindo anteriores, os fatores associados foram o sexo feminino (OR = 1,19; IC95% 1,00–1,42), renda menor que R$ 1.500,00 (OR = 1,65; IC95% 1,33–2,05) e entre R$ 1.500,00 e 2.500,00 (OR = 1,82; IC95% 1,47–2,25), escolaridade menor que 5 anos (OR = 2,2; IC95% 1,68–2,90) e entre 5 e 9 anos (OR = 2,01; IC95% 1,65–2,45) e percepção positiva sobre a necessidade de tratamento (OR = 1,95; IC95% 1,57–2,43), além de procura do serviço por rotina (OR = 1,83; IC95% 1,50–2,24), motivada por dor (OR = 3,49; IC95% 2,46–4,97) e para extração dentária (OR = 1,97; IC95% 1,53–2,55). Para os que perderam acima de 12 dentes, foram o sexo feminino (OR = 1,42; IC95% 1,09–1,86), renda menor que R$ 1.500,00 (OR = 1,63; IC95% 1,13–2,33) e entre R$ 1.500,00 e 2.500,00 (OR = 1,97; IC95% 1,37–2,83), escolaridade menor que 5 anos (OR = 7,19; IC95% 5,02–10,32) e entre 5 e 9 anos (OR = 4,71; IC95% 3,49–6,37) e procura do serviço por rotina (OR = 4,09; IC95% 2,70–6,17), dor (OR = 11,79; IC95% 7,02–19,80) e extração dentária (OR = 3,60; IC95% 2,25–5,77).

**Tabela 3:** Análise bruta dos fatores associados segundo a classificação da perda dentária em adultos de 35 a 44 anos. Levantamento epidemiológico de saúde bucal do estado de São Paulo, 2015.

Variáveis		Classificação da perda dentária								
		Perdeu até 12 dentes posteriores			Perdeu até 12 dentes, incluindo anteriores			Perdeu acima de 12 dentes		
		OR bruto	IC95%	p-valor	OR bruto	IC95%	p-valor	OR bruto	IC95%	p-valor
Determinantes primários de saúde										
Sexo	Feminino	1,14	0,99–1,32	0,016	1,28	1,11–1,47	0,001	1,46	1,20–1,78	< 0,001
	Masculino	1,00			1,00			1,00		
Renda familiar	Baixa	1,68	1,40–2,01	< 0,001	2,71	2,25–3,24	< 0,001	4,31	3,25–5,71	< 0,001
	Média	1,86	1,55–2,23	< 0,001	2,22	1,83–2,68	< 0,001	2,81	2,08–3,78	< 0,001
	Alta	1,00			1,00			1,00		
Escolaridade	< 5 anos	1,54	1,20–1,98	0,001	3,25	2,56–4,11	< 0,001	13,22	9,91–17,63	< 0,001
	5–9 anos	1,57	1,32–1,87	< 0,001	2,64	2,23–3,13	< 0,001	6,44	5,05–8,23	< 0,001
	> 9 anos	1,00			1,00			1,00		
Número de pessoas morando na mesma casa	< 3 pessoas	1,18	1,03–1,36	0,018	1,26	1,10–1,45	0,001	1,20	0,99–1,45	0,052
	> 3 pessoas	1,00			1,00			1,00		
Percepção da necessidade de tratamento	Não	1,69	1,45–1,98	< 0,001	3,02	2,54–3,58	< 0,001	1,96	1,56–2,46	< 0,001
	Sim	1,00			1,00			1,00		
Tipo de serviço	Público	1,4	1,20–1,63	< 0,001	1,44	1,24–1,66	< 0,001	1,77	1,45–2,15	< 0,001
	Convênio	1,33	1,05–1,68	0,015	1,33	1,05–1,68	0,497	0,67	0,46–1,0	0,051
	Particular	1,00			1,00			1,00		
Avaliação do tratamento	Não boa	1,57	1,26–1,96	< 0,001	1,75	1,41–2,16	< 0,001	1,79	1,36–2,35	< 0,001
	Boa	1,00			1,00			1,00		
Comportamentos de saúde bucal										
Tempo desde a última consulta	< 1 ano	1,28	1,04–1,56	0,016	1,48	1,21–1,80	< 0,001	2,84	2,23–3,60	< 0,001
	1–2 anos	1,19	1,01–1,41	0,034	1,24	1,05–1,46	0,011	1,28	1,01–1,62	0,039
	> 2 anos	1,00			1,00			1,00		
Motivo da consulta	Rotina	1,55	1,32–1,83	< 0,001	2,48	2,08–2,95	< 0,001	5,08	3,68–7,00	< 0,001
	Dor	2,41	1,78–3,26	< 0,001	5,11	3,81–6,88	< 0,001	21,55	14,45–32,13	< 0,001
	Extração	1,88	1,52–2,34	< 0,001	3,39	2,72–4,22	< 0,001	6,34	4,40–9,16	< 0,001
	Tratamento	1,00			1,00			1,00		
Resultados de saúde bucal										
Bolsa periodontal	Sim	1,24	1,05–1,47	0,012	1,96	1,67–2,30	< 0,001	2,44	1,96–3,03	< 0,001
	Não	1,00			1,00			1,00		
Cárie dentária	Sim	1,31	1,14–1,50	< 0,001	1,97	1,69–2,30	< 0,001	1,82	1,49–2,24	< 0,001
	Não	1			1			1,00		
Dor de dente	Sim	1,32	1,12–1,55	0,001	1,97	1,69–2,30	< 0,001	1,83	1,49–2,24	< 0,001
	Não	1,00			1,00			1,00		

**Tabela 4:** Análise ajustada dos fatores associados segundo a classificação da perda dentária em adultos de 35 a 44 anos. Levantamento epidemiológico de saúde bucal do estado de São Paulo, 2015.

Variáveis		OR ajustado	IC95%	p-valor
Perdeu até 12 dentes				
Sexo				
	Feminino	1,11	0,94–1,31	0,204
	Masculino	1,00		
Renda	< R$ 1.500,00	1,39	1,13–1,71	0,002
	R$ 1.500,00–2.500,00	1,78	1,46–2,17	< 0,001
	> R$ 2.500,00	1,00		
Escolaridade	< 5 anos	1,28	0,96–1,69	0,088
	5–9 anos	1,39	1,14–1,70	0,001
	> 9 anos	1,00		
Percepção da necessidade de tratamento	Sim	1,36	1,12–1,65	0,002
	Não	1,00		
Motivo da consulta	Rotina	1,30	1,08–1,56	0,006
	Dor	2,04	1,43–2,90	< 0,001
	Extração	1,37	1,07–1,75	0,013
	Tratamento	1,00		
Avaliação do serviço	Bom	1,33	1,04–1,70	0,024
	Não bom	1,00		
Tipo de serviço	Público	1,17	0,98–1,39	0,080
	Convênio	1,39	1,07–1,80	0,012
	Particular	1,00		
Bolsa periodontal	Sim	1,05	0,86–1,28	0,652
	Não	1,00		
Cárie dentária	Sim	1,01	0,85–1,20	0,878
	Não	1,00		
Perdeu até 12 dentes, incluindo anteriores				
Sexo	Feminino	1,19	1,00–1,42	0,044
	Masculino	1,00		
Renda	< R$ 1.500,00	1,65	1,33–2,05	< 0,001
	R$ 1.500,00–2.500,00	1,82	1,47–2,25	< 0,001
	> R$ 2.500,00	1,00		
Escolaridade	< 5 anos	2,21	1,68–2,90	< 0,001
	5–9 anos	2,01	1,65–2,45	< 0,001
	> 9 anos	1,00		
Percepção da necessidade de tratamento	Sim	1,95	1,57–2,43	< 0,001
	Não	1,00		
Motivo da consulta	Rotina	1,83	1,50–2,24	< 0,001
	Dor	3,49	2,46–4,97	< 0,001
	Extração	1,97	1,53–2,55	< 0,001
	Tratamento	1,00		
Avaliação do tratamento	Bom	1,30	1,02–1,67	0,036
	Não bom	1,00		
Tipo de serviço	Público	0,97	0,81–1,16	0,764
	Convênio	1,17	0,89–1,54	0,269
	Particular	1,00		
Bolsa periodontal	Sim	1,33	1,10–1,62	0,004
	Não	1,00		
Cárie dentária	Sim	1,35	1,13–1,61	0,001
	Não	1,00		
Perdeu acima de 12 dentes				
Sexo	Feminino	1,42	1,09–1,86	0,010
	Masculino	1,00		
Renda	< R$ 1.500,00	1,63	1,13–2,33	0,008
	R$ 1.500,00–2.500,00	1,97	1,37–2,83	< 0,001
	> R$ 2.500,00	1,00		
Escolaridade	< 5 anos	7,20	5,02–10,32	< 0,001
	5–9 anos	4,71	3,49–6,37	< 0,001
	> 9 anos	1,00		
Percepção da necessidade de tratamento	Sim	1,81	1,25–2,60	0,001
	Não	1,00		
Motivo da consulta	Rotina	4,09	2,70–6,17	< 0,001
	Dor	11,79	7,02–19,80	< 0,001
	Extração	3,60	2,25–5,77	< 0,001
	Tratamento	1,00		
Avaliação do serviço	Bom	1,12	0,78–1,61	0,521
	Não bom	1,00		
Tipo de serviço	Público	0,96	0,73–1,24	0,733
	Convênio	0,75	0,45–1,24	0,266
	Particular	1,00		
Bolsa periodontal	Sim	1,44	1,10–1,90	0,009
	Não	1,00		
Cárie dentária	Sim	1,15	0,88–1,51	0,296
	Não	1,00		

Nota: A categoria de referência para a análise de regressão multinomial foi “não ter perdido nenhum dente por cárie ou doença periodontal’’.

## DISCUSSÃO

No presente estudo, a menor renda e escolaridade, a percepção de necessidade de tratamento e última consulta motivada por rotina, dor ou extração foram associados à perda dentária, independentemente da classificação. A avaliação negativa do serviço odontológico foi associada aos indivíduos que perderam até 12 dentes, tanto anteriores quanto posteriores. O sexo feminino e a presença de bolsa periodontal foram associados a perdas dentárias de até 12 dentes, incluindo anteriores, e acima de 12 dentes. A presença de cárie foi associada apenas aos indivíduos que perderam até 12 dentes, incluindo anteriores.

Na literatura, são encontrados estudos que avaliam as perdas dentárias de forma numérica, utilizando como base de corte a presença de 20 dentes ou mais^7^. Verifica-se que o uso da classificação das perdas dentárias proposta por Batista et al.^8^foi capaz de mensurar fatores associados de forma mais específica conforme o número e a posição dos dentes no arco dental. Ela baseia-se na teoria do arco dentário reduzido, considerando satisfatória a presença de dez pares de dentes ocludentes sem lacunas estéticas^15^e considerando perdas dentárias por cárie e doença periodontal, excluindo dentes ausentes congenitamente ou por motivos ortodônticos, o que já era critério da OMS^11^. Clinicamente, com a incorporação de dentes perdidos por doença periodontal, essa nova classificação permite identificar a questão estética e funcional, importantes também para o planejamento da reabilitação oral dos pacientes. Além disso, em estudo prévio, verificou-se que a posição e número de dentes perdidos apresentam impactos diferentes na qualidade de vida dos adultos^7^.

A literatura demonstra que diversos estudos têm associado as perdas dentárias com menor renda e escolaridade^16 , 17 , 18 , 6 , 19^. Isso pode ser explicado pelo fato de os indivíduos mais pobres e menos escolarizados residirem em localidades com menor cobertura de fluoretação de águas^20^, terem menos acesso a serviços odontológicos^21 , 4^e produtos de higiene pessoal^4^e praticarem hábitos inadequados, como consumir mais açúcar^22^e escovar os dentes com menos frequência^23^.

Dados da Pesquisa Nacional por Amostra de Domicílios (PNAD) mostraram que indivíduos com maior nível de instrução apresentam maior frequência em consultas odontológicas: 67,4% entre aqueles com nível superior completo e 36,6% entre pessoas sem instrução ou com ensino fundamental incompleto^24^. Para Chrysanthakopoulos^25^, a escolaridade também tem demonstrado um impacto sobre a capacidade de auto percepção dos indivíduos sobre o estado da sua condição de saúde bucal e a avaliação da necessidade de tratamento odontológico. No presente estudo, foi verificada uma associação entre maior grau de escolaridade e menor o número de dentes perdidos; no entanto, a percepção de necessidade de tratamento também foi associada às perdas dentárias, independentemente da classificação. O estudo de Santillo et al.^26^em uma população rural de Pernambuco (PE) encontrou relação entre a perda dentária e a auto percepção negativa sobre a saúde bucal. Esses resultados indicam que o modelo de odontologia ainda tem um caráter mutilador e que a percepção da necessidade de tratamento é constatada apenas em estágios avançados das doenças bucais, determinando a busca tardia por serviços odontológicos^27 , 28^.

Essa ideia pode ser reforçada no presente estudo, em que a última consulta odontológica motivada por rotina, dor ou extração esteve associada com as perdas dentárias, independentemente da classificação. Apesar de a literatura relacionar a procura do serviço odontológico por dor com a prevalência^8 , 29^e incidência de perdas dentárias^5^, este é provavelmente o primeiro estudo que mostra também associação com procura do serviço para extração de dente e por rotina. Esse resultado pode ser explicado pelo histórico de atenção à saúde bucal brasileira, principalmente na faixa etária de adultos, marcado pela restrição de acesso e alta demanda^4^.

Além disso, a busca tardia pelos serviços de saúde bucal determina a progressão das doenças bucais, acarretando a necessidade de procedimentos e técnicas mutiladoras e especialmente a perda dos dentes^6 , 28 , 30^. Outro aspecto relevante é como tornar a busca pelos serviços odontológicos rotineira em uma população economicamente ativa. O estudo de Silva-Junior et al.^28^verificou que a escolha por extrair os dentes ao invés de mantê-los se deve principalmente à ausência de outra opção de tratamento no momento da consulta e ao alto custo dos procedimentos necessários à manutenção dos dentes.

Outro aspecto relevante para este estudo foi a associação entre a classificação das perdas dentárias e a avaliação dos serviços odontológicos. A avaliação dos serviços de saúde pelos usuários auxilia na construção de indicadores visando à implementação de estratégias de saúde do serviço, direcionando as ações de prevenção e promoção^31^. Um estudo realizado na Bahia verificou desigualdade na utilização de serviços de saúde bucal mesmo entre os usuários do serviço público em diferentes níveis assistenciais. Aqueles que tinham menor escolaridade e que estavam expostos a uma pior organização do serviço usaram menos o serviço^32^.

Quando comparado à assistência suplementar, o serviço público apresentou um perfil de vulnerabilidade, embora a desorganização do serviço não favoreça o seu uso. Os resultados reforçam que o enfrentamento das desigualdades no acesso e utilização de serviços públicos de saúde é dependente do modo como o governo local articula o projeto e sua capacidade de reorganizar a atenção odontológica. Sendo assim, é necessário pensar em modos de promover o uso e satisfação com os serviços públicos de saúde, principalmente em adultos. Essa faixa etária economicamente ativa enfrenta a restrição do horário de funcionamento das unidades de saúde, o que dificulta o seu acesso e consequentemente a resolutividade de estágios iniciais das principais doenças bucais.

No presente estudo, houve associação das condições clínicas de saúde bucal, cárie e doença periodontal, com as perdas dentárias entre indivíduos que mantêm maior quantidade de dentes posteriores. A partir desse achado, pode-se inferir que a presença de dentes posteriores aumenta a ocorrência da cárie e doença periodontal, o que explicaria a manutenção dessa associação até no modelo ajustado final. Os dentes posteriores são os mais acometidos pelas doenças bucais^5^, principalmente pelo desconhecimento da presença de dentes permanentes ainda na infância e por estarem em áreas não estéticas, o que dificulta visualizar a necessidade de tratamento na ausência de sintomatologia dolorosa e prorroga a procura do serviço odontológico para tratamento.

Isso pode ser evidenciado no nosso estudo, pois a variável de auto percepção da necessidade de tratamento foi associada às três categorias de perdas dentárias. Por isso, indivíduos que perderam até 12 dentes, incluindo anteriores, e que, portanto, ainda mantêm dentes posteriores, estiveram associados à presença de cárie e bolsa periodontal, e as perdas acima de 12 dentes apenas à bolsa periodontal. O estudo de Batista et al.^8^também verificou associação entre indivíduos que perderam 12 dentes, incluindo anteriores, com bolsa periodontal. Sendo assim, estudos que levam em consideração a posição dos dentes perdidos podem inferir de forma mais fidedigna a associação de variáveis para a perda dentária.

Dannewitz et al.^33^demonstraram que o acesso ao tratamento da terapia periodontal resulta em um bom prognóstico dos molares. Segundo os resultados demonstrados neste estudo, a presença de bolsa periodontal não apresentou associação com quem já perdeu dentes posteriores. A escassez do acesso a serviços especializados para realização de tratamento periodontal pode contribuir para o grande número de perdas dentárias, principalmente dos molares. Mesmo com o lançamento, em 2004, da atual Política Nacional de Saúde Bucal e a consequente ampliação da oferta de serviços odontológicos especializados, o número de procedimentos periodontais e endodônticos não aumentou em todos os municípios estudados. Isso se deve a uma série de possibilidades e justificativas, tais como a disponibilidade da força de trabalho e sua distribuição geográfica, a facilidade ou não de acesso a serviços odontológicos, as características da gestão do serviço ou organização da rede assistencial e do processo de trabalho^34^. Ressalta-se que a política ainda é recente, principalmente as ações da atenção secundária.

No presente estudo, observou-se que o principal componente na experiência de cárie entre os adultos foram os dentes restaurados. Esse dado também foi verificado no último levantamento nacional de saúde bucal nas regiões Sul e Sudeste do país, que apresentam melhores condições socioeconômicas, e pode refletir uma maior resolutividade em estágios iniciais da cárie dental e incorporação de tratamentos menos invasivos^4^, assim como o impacto da inserção e expansão da Política Nacional de Saúde Bucal. O diagnóstico das condições de saúde bucal e das necessidades de tratamento da população, bem como a avaliação do modelo de atenção em saúde vigente, é de fundamental importância como primeiro passo para a programação e planejamento em saúde bucal, permitindo estabelecer prioridades de ação e alocação de recursos de forma direcionada à melhoria das condições de saúde da população^35^.

As limitações do estudo estão no seu caráter transversal, com exposição e desfecho avaliados em um único momento no tempo, podendo haver viés de prevalência, além de uma maior participação de mulheres por ser uma pesquisa domiciliar com adultos. Além disso, o questionário contemplava informações sobre experiências pregressas de atendimento odontológico, as quais dependem da memória do indivíduo para sua exatidão.

As categorias da nova classificação da perda dentária, considerando a posição e o número de dentes perdidos, permitiram identificar diferentes fatores associados. Os resultados do nosso estudo apontam uma evidente necessidade de se considerar uma avaliação qualitativa e quantitativa das perdas dentárias, para que elas não sejam subestimadas, e uma importante observação a ser levada em conta em estudos futuros, inclusive para subsidiar tomadas de decisão na oferta e organização de serviços odontológicos.
